# The experience of engaging with mental health services among young people who hear voices and their families: a mixed methods exploratory study

**DOI:** 10.1186/s12913-014-0527-z

**Published:** 2014-11-05

**Authors:** Prerna Kapur, Daniel Hayes, Rachel Waddingham, Saul Hillman, Jessica Deighton, Nick Midgley

**Affiliations:** University College London and Anna Freud Centre, 12 Maresfield Gardens, London, NW3 5SU UK; Mind in Camden, Barnes House, 9-15 Camden Road, London, NW1 9LQ UK

## Abstract

**Background:**

Research shows us that auditory hallucinations or ‘hearing voices’ may be more common than previously thought, particularly in childhood and adolescents. Importantly, not all individuals are affected negatively by their voice hearing experiences, yet child and adolescent mental health services (CAMHS) have traditionally understood voice hearing as a symptom of psychosis and severe mental illness, with implications for the way interventions are offered. The purpose of the present study was to gain an understanding of how young people who hear voices and their families find engaging with mental health service, and to better understand their experience of mental health professionals.

**Methods:**

A two-stage, mixed methods study was used. In the first stage, semi-structured interviews were carried out with two young people and their parents who had engaged with mental health services, and the collected data were analysed using Interpretative Phenomenological Analysis (IPA). In the second stage, a questionnaire was designed to test the generalizability of the themes arising from the first stage, and was completed online by 32 young voice hearers and 27 parents.

**Results:**

IPA analysis produced 4 themes: (1) The struggle to understand the hearing voices phenomenon; (2) Battle with the Mental Health Services; (3) ‘Stuck in a limbo’; and (4) The wish for a more holistic approach from mental health services and professionals. The survey partially confirmed the findings of study one, with young people and parents finding useful information difficult to come by, and many reported feeling lost in CAMHS. Additionally, young voice hearers and parents often felt not listened to, and many parents expressed the need for a holistic care, whilst young people wanted a more normalizing and less stigmatizing experience.

**Conclusions:**

Young people and their families had varying experiences of mental health services. Whilst the survey showed that some young people and their families had more positive experiences, many expressed dissatisfaction. To fulfil the needs of young people and their families, mental health services would benefit from developing alternative approaches to voice hearing and running support groups that could form part of a ‘normalising’ and ‘holistic care’ package.

**Electronic supplementary material:**

The online version of this article (doi:10.1186/s12913-014-0527-z) contains supplementary material, which is available to authorized users.

## Background

‘Hearing voices’ is a term commonly used to describe auditory hallucinations. Whilst voice-hearing was once thought to be an indicator of severe mental illness, most notably psychotic disorders [1-4]; epidemiological evidence suggests that these experiences are relatively common in childhood and adolescence, with prevalence rates between 5.7 and 21% [5-9]. Despite this, the understanding of hearing voices in a non-medical context remains an unresolved issue in psychiatry and clinical care [10].

Attitudes towards, and explanations for, voice hearing have changed over time [11]. Traditionally, action taken by professionals in response to voice hearing would be to pathologise such experiences, prescribe antipsychotic medication, and see such individuals as ‘patients’ or ‘sufferers’ [11,12]. In light of voice hearing being more common that previously thought [5-9], and that such individuals are not always adversly affected by their experiences [13], it has been proposed that alternative explanations to voice hearing should be examined [12].

One such explanation is to put a greater focus on the lived experience of the voice hearer. This has been reflected in the Hearing Voices Movement’s prioritisation of an individual’s right to define their own experiences [14]. But although establishing meaning in a voice hearer's experiences has been shown have a positive impact on voice hearers, biological paradigms still continue to dominate explanations of voice hearing [11]. Coffey and Hewitt [15]show clear differences between help being sought by voice hearers, and help being provided by community mental health nurses; with voice hearers experiencing the help given as being highly medical in nature, even if Community Mental Health Nurses considered themselves to be taking the social context into consideration and not looking at the individual in isolation.

Thus far, the vast majority of research into people’s experiences of voice-hearing and mental health care has focused on adults, with a small but growing interest in the experiences of clients involved with Early Intervention in Psychosis Services (EIS). Research is also needed into the experience of children who report hearing voices to professionals in child and adolescent mental health services (CAMHS). In one of the only studies specifically examining the experiences of young people (16–25 year olds) who hear voices in EIS, Bampton [16] found that psychosis was often the account given for why the young person was hearing voices, with biomedical explanations more likely to be validated by professionals than other explanations. Despite this, some research suggests a considerable interaction between dispositional and situational factors where voice hearing is concerned [17]. For example, Escher and colleagues [18] point out that voice hearing ceases in some cases when a child’s emotional or situational difficulties are solved. Thus accessing early support, be that NHS or otherwise, may be of benefit for individuals that hear voices. However, given the stigma and discrimination that are frequently associated with the experience of voice hearing [12,19,20] young people may fear disclosure, instead choosing to hide their experiences [21].

In summary, whilst there has been emerging literature into new approaches to voice hearing and putting such approaches into practice, very little literature has concentrated on how children and young people experience voice-hearing, and even less about how they and their families experience mental health services. As such, the aim of this study was to gain an insight into the experiences and perspectives of young people (aged 11–18) who hear voices and their families who have engaged with mental health professionals, and to examine their views on such services.

## Method

### Design

The study was conducted in two parts. In the first stage, semi-structured interviews were conducted with 4 individuals (2 young voice hearers and 2 parents) on a one to one basis, to gain an in-depth understanding of these families' experiences of engaging with mental health services. In the second stage, a survey was formulated based on the findings from the first stage of the study. The aim of the second stage was to try and explore whether the themes found through the in-depth interviews could be extended or were applicable to a relatively larger and varied population.

### Participants and collection of data

Prior to data collection, ethical permission was sought and granted from University College London Research Ethics Committee. Participants were young people (aged 11–18) who hear voices and their families. The lower limit was kept to 11 as a minimal level of communicative and comprehensive skills were required to take part in this research. For stage one (interviews), participants were recruited through Voice Collective; a London-wide project set up to support children and young people who hear voices, see visions or have other unusual sensory experiences; as well as providing support and education to their families and professionals. Individuals were invited to take part either via the project manager, or could sign up via the Voice Collective website (www.voicecollective.co.uk).

Through this processes, two young people and their parents expressed an interest in taking part and informed consent (or assent and informed consent for a parent/guardian for those under 16) was obtained after participants had read the information sheet, and were given the opportunity to ask any questions. Background information about the two parent–child dyads is outlined below (pseudonyms have been used to maintain anonymity):

1. Linda: An 11 year old Caucasian female, and her mother Sasha, who was in her 40’s. It was reported via Sasha that Linda had been hearing voices since the age of seven, and found these voices distressing as they were telling her to do things that she didn’t want to do. Linda had intermittent contact with CAMHS and was diagnosed with Reactive Attachment Disorder at 10. Linda has been taking antipsychotic medication since nine, and was admitted to an inpatient ward at the age of 10 due to voice hearing. At the time of interviewing, Linda was still hearing voices and had been engaging in self-harm.

2. Andy: A 17 year old Caucasian male, and his mother Louise who was in her 40’s. It was reported via Louise that Andy had been hearing voices since 12, and found this distressing. At 14, Andy was referred to CAMHS,and later was admitted to an inpatient ward. Previous diagnoses include Myalgic Encephalomyelitis and Psychosis. Andy had been offered mood stabilising medication, but with his mother’s permission decided not to take them.

For the second stage of the study, 32 young voice hearers and 27 parents responded to an online survey, Additional file 1 and Additional file 2 show these surveys in more detail. This was posted on the Voice Collective and Intervoice websites (www.voicecollective.co.uk and www.intervoiceonline.org) along with an invitation to participate, explaining that the survey aimed to find out about the experiences of young people who hear voices and their families about getting help and support for these experiences, and the way they felt about mental health professionals. It was made clear that the survey was based on in-depth interviews with a small number of families, and that the survey aimed to find out whether their experiences are similar to, or different from, a wider group.

The young people who responded to the survey ranged in age from 12–29 (mean: 17.7 years), and included 26 girls and six boys. Respondents were mainly from the UK (n = 13) and the USA (n = 10) though other responses were from Canada, Brazil, Serbia and New Zealand. The parents who completed the survey (it is not known whether some of these were parents of the young people who also completed the survey) ages ranged from 31 to 57 years (mean: 45.4 years), and included 25 mothers and two fathers. Parents came from the USA (n = 14), the UK (n = 9), Canada, Australia, Mexico and Greece, and had voice-hearing children ranging in age from five to 24 years (mean: 14.8 years), of which 15 were female and 12 male.

### Materials/Interview construction

The semi-structured interview schedule for stage one of the study covered a range of issues. History of help-seeking from mental health services, current mode of support, expectations and anxieties related to mental health services and professionals, and experience of services were identified as key areas of interest. Questions in relation to each identified area were formulated along with possible probes and prompts, but the interview schedules were used flexibly in order to ensure that the process was led by the participants [22].

Based on the findings of the interviews, two separate surveys about the experience of Child and Adolescent Mental Health Services (CAMHS) among young people who hear voices and their parents were constructed. The surveys consisted of questions relating to knowledge of voice hearing, accessibility of services, inclusion in care choices, and information around feelings and experience of services and voice hearing.

### Data analysis

The data from the semi-structured interviews was analysed using Interpretative Phenomenological Analysis (IPA), a qualitative methodology which is widely used in the field of psychology and mental health [23]. The interviews were first transcribed verbatim and then read several times with emergent themes documented. Analysis was first undertaken by the primary researcher (PK), and then along with supervisors (NM, SH) to minimise idiosyncratic or pre-determined themes from emerging. The researchers then attempted to individually group the agreed themes into clusters, before coming up with a final cluster of themes for each interview. Cluster of themes from each interview were then generated into superordinate themes that attempted to encapsulate the essence of the experience of the participants. This approach allows the researcher to play a dynamic role and make sense of the interviewees’ lifeworlds using a ‘double hermeneutic’, i.e. the researcher’s meaning-making in relation to the participant’s own sense-making of their personal experience [22]. Once the interviews were analysed, a summary capturing the essence of their experience through the emergent themes was emailed to the participants. This gave the participants a chance to feedback on the results; such feedback may be considered as ‘respondent validation’ [22]. Only Linda responded, but stated that she felt the themes were an excellent fit of her experiences of CAMHS.

For the second stage of the study, questionnaire responses were analyzed using descriptive statistics. The results from the surveys were then viewed in light of the themes that emerged from the interviews and their relationship was examined.

## Results

### Stage 1: Interviews

Four superordinate themes emerged from the interviews which are presented below in narrative form. Although not all themes were found in all four interviews, we have indicated where a theme was specific to a particular participant.

1.The struggle to understand the hearing voices phenomenon

Both young people and parents spoke of a ‘struggle’ with understanding the experience of ‘hearing voices’. Andy (17) described this below:

***‘****We went to various different people, and asked them. And I wasn’t sure what was going on. And I was quite scared. And several things happened in my life at once,…..it was at that point that like things started to get really bad and I started to get really depressed and started to get really worried.’*

Interviews with the parents suggested that they looked for meaning in terms of a biological-psychological interaction and developmental explanations. Despite this, they were left feeling that they had no definitive answers. Sasha illustrated this:

*‘I look back so much at that time to see what made it change. I will never know … because it all coincided around the same time, whether that…her bodily changes and stuff, I don’t know! Whether it was all too much? Whether the hormones on a developing brain caused something? I will probably never know! I would love to have, you know, a definitive answer, but I don’t know if I’m ever going to have one now.’*

It appears that this persisting uncertainty about the understanding of ‘hearing voices’ was a primary reason why these families turned to CAMHS as a source of information and knowledgeable support.

2. Battling with the Mental Health Services

All four interviewees expressed scepticism about the effectiveness of available mental health services, based on their own experiences. Young people felt that there was a distance between them and the CAMHS professionals. Linda outlined this below:

*‘I think in the beginning when I was attending the appointments at [CAMHS], I felt like I was wasting their time. So I felt like in the beginning they didn’t believe me. So I feel like they were kind of annoyed at me as I was wasting their time. As I got to gradually get to know them, I kind of realized that they do kind of understand a bit more.’*

Andy also reports felling distanced from mental health professionals:

*‘There always seems, even when I was in hospital that someone, it seemed it happened that someone was more deserving of services than I was. And that I wasn’t, not worthy..not that I wasn’t worthy…that I wasn’t worth as much time as other people were.’*

Thus, the perceived feelings of isolation from CAMHS professionals led young people to feel that they were less ‘deserving’ of the mental health professionals time and resources. Parents also felt at odds with professionals; however they expressed this in terms of ‘not being heard’, and feeling under-supported. Louise explained:

*‘I think they see us in a slightly ambiguous way. On the one hand, we are seen as a really important source of support for - for Andy. But on the other hand we are also seen as. I think a sort of irritation and an annoyance… But then - because they have no psychological resources, they then rely on - they want Andy to be in the community. Who’s going to look after him in the community most? It’s going to be us! And they don’t actually then give us, really any help very much in looking after him in the community… the level of help that the community team is able to provide seems very limited. It seems limited to medication and psychiatric oversight. Umm, and the team doesn’t seem to have a psychologist, it doesn’t seem to have any of the sort of talking therapy support that I think is essential for somebody with Andy’s circumstances.’*

Based on these findings, it may seem that having access to resources in order to facilitate care, and also psychological resources, may influence one's experience of CAMHS.

3. ‘Stuck in a limbo’

Both young people and their parents expressed frustration around CAMHS care received (in terms of consistency, quality and speed). Andy described it like this:

*‘It made me feel like I was kind of being passed around. Like umm…not…really….well, that’s not necessarily true. I didn’t feel like I wasn’t cared for. Well I did, for a while. Until I started well seeing people like Sarah [name changed], who is a psychologist who does CBT with me sometimes. And she’s very nice and she makes me feel like I’m cared for. But like, before like, I kind of felt like no one had enough time for me and I was just passed on. When people thought there was nothing else they could do for me.’*

Andy’s narrative shows that he wanted CAMHS professionals to have enough time and patience for him; once he was able to find these qualities in someone, he was able to form a therapeutic relationship with them, which made him feel looked after. Linked in with this, it appears that to get the attention of the professionals, Louise and Andy both felt that they had to have a certain level of severity within voice hearing. If their need didn’t seem urgent, they felt like they were a ‘waste of time’.

Whilst both parents and the young voice hearers found that explaining the meaning of voices and understanding them in developmental contexts beneficial, they felt such services were limited and inaccessible. For them, CAMHS did not provide enough psychological support, especially in terms of alternative ways of dealing with voices. Neither parent nor young person seemed very hopeful about the treatment they were receiving. For Sasha, this is detailed by the escalation in Linda’s care from not getting better or reacting well to any of the antipsychotic medication, to ending up being admitted to hospital, again with no option of psychological input:

*‘So, in the end she [Linda] was pleading with me not to continue with the medication, So, we stopped. So then they tried her on another medication. Again she had really significant side-effects. So they decided that he felt she needed to be admitted to a hospital. Linda was, I mean again she was not allocated a psychologist on admission. And I kind of assumed part of it would be for her to have a psychological input.’*

4. Wish for a holistic approach

Young people and parents hoped for a more all-inclusive approach that would provide a space to share and express themselves without feeling judged. Linda expressed this with the following:

*‘I think the good side would have to be doctors who are understanding … And supportive. Umm, welcoming. Umm, I think they would have to have like, because when they like read from their books, like all those longer words that younger people don’t understand, so I think they need to have like children social skills.’*

***Interviewer:****‘****What would you want to feel like? If you want some special kind of service?’***

*‘I think to feel comfortable being in the room talking to them. And like feel like they are listening to you and not just like being annoyed and feeling like you’re a waste of time. So I think to feel comfortable.’*

Places where young people and parents with similar experience could come together were also considered extremely helpful. They conveyed their belief that such a setup could bring a sense of belonging and shared experience that other services were not able to provide. Louise also gives high regard to professionals who have had experiences of ‘hearing voices’ and opportunities for peer support, as it may bring a sense of identification and empathy. For Louise, this sense of mutual understanding tends to de-stigmatize hearing voices as an ‘illness’:

*‘That’s one source of support [peer group support] now that is open to Andy where he can actually meet people who have similar experiences to him and get support from them which is fantastic. He can’t get that support on the NHS. Or everybody who treats and deals with a young person like Andy doesn’t have the understanding of what it’s like, doesn’t understand the experiences, has a particular sort of a view of mental illness that’s about it being an illness and about it being treated by medication primarily.’*

While talking about an ideal service, both parents mentioned the importance of a multi-faceted healthcare approach that would extend beyond the child’s immediate medical needs and focus on the social interactions, adjusting and coping of the young person in ordinary life.

Sasha explained:

*‘I’d also make it more of a holistic approach as well, so that they’re looking at … resolving other issues within the family and within the environment as well and looking at, just supporting the family as a whole. I just wish that service provided the psychotherapist, the psychologist, the psychiatrist all under within that kind of… the social workers… whatever else is there that is required, all within that same kind of umbrella..’*

Both Louise and Sasha also express a wish for specialist support for parents of young people who hear voices, which would primarily focus on their particular needs.

### Stage 2: Surveys

32 young people and 27 parents responded to the surveys designed to test out the generalizability of the themes found in the first stage of the study. The results of the survey are presented below.

1) Knowledge/Information

Nearly two-thirds of young people (21/30) had some certainty about the reasons they heard voices with 23% of the total sample saying they felt ‘very sure’ about the reasons they heard voices. Though most of them had some notion of where the ‘voices’ came from, 30% (nine young people) did not know why they heard these voices. In the parallel survey carried out with parents, a similar but even higher percentage, 78%, (21/30 parents) shared some level of certainty about why they heard voices. Interestingly, a much lower percentage (11%) had absolute certainty about this. Again, a similar but lower percentage (22%) did not know why their child heard voices.

When young people were asked about how they found accessing ‘useful’ information to aid their understanding of voice hearing, most young people found this ‘quite difficult’ or ‘very difficult’. Indeed, only 13% (4/30) found the information easy to access, with the remaining 26 young people finding it either ‘quite’ or ‘very’ difficult. In the parallel parental survey, an almost identical pattern emerged with an overwhelming majority of parents finding it difficult to access information about such experiences to aid their understanding. Interestingly, parents were even more likely to perceive the information as ‘very difficult’ rather than ‘quite difficult’.

2) Feelings

There was a diverse range of feelings that young people expressed in relation to hearing voices. In the parental survey, there was a similar spread of emotional states. Below, the most prevalent feelings are reported.

Confusion: 83% (25/30) of young people talked about feeling ‘confused’ and this was the strongest choice. In the parents’ survey, this was experienced but to a lesser degree (52%).

Anxiety and Stress: Two thirds, 67% (20/30) of young people talked about feeling ‘anxious’, 57% (17/30) felt ‘scared’, and 47% (14/30) felt ‘stressed’. Interestingly, in the parent survey, feeling ‘anxious’ and ‘scared’ were the most common feelings that they felt about having a child who heard voices: 70% (19/27) and 67% (17/27) respectively responded this way. Higher rates of stress compared to young people were also experienced by the parents (63%).

Anger: 40% (12/30) of young people talked about feeling ‘angry’ about their experience. A slightly smaller proportion of parents in the parallel survey (30%) experienced this.

Lonely: 63% (19/30) of young people talked about feeling ‘lonely’ as a result of their experience. A further 57% (17/30) described themselves as feeling ‘lost’. In the parental survey, a much lower number of parents, 33% felt ‘lonely’ as a result of having a child with such a disorder, whilst a very similar proportion of 52% (14/27) felt ‘lost’ as a result.

Crazy: A high proportion of nearly half (14/30) of young people talked about feeling ‘crazy’ about their experience.

3) Access to Help

Just over a quarter (8/30) of young people felt it had been ‘quite’ or ‘very’ easy to access help, whilst over half, 53% (16/30) had found the experience difficult in some way. The remaining 20% (6/30) stated that they had never tried to get help from the mental health services. In the parental survey, a similar spread of responses was found with 30% (8/27) finding it ‘quite’ or ‘very’ easy to access help with a high proportion of 59% (16/27) finding this difficult. A much smaller percentage (11%) stated they had not previously had to contact mental health services.

Similarly, young people varied in their responses as to how much they thought professionals involved in their care took their ideas, wishes and views into account. Only 20% (6/30) felt that this had been done ‘a lot’ whilst a third (10/30) felt this had been partially addressed. A relatively large proportion (47%) felt that was not considered ‘much’ or ‘at all’. In the parental survey, the patterns were slightly different with a greater percentage of parents (33%, 8/24) saying their ideas and wished had been considered ‘a lot’ and a further 29% (7/24) ‘a little’. Overall, a smaller percentage of parents felt professionals had not taken their thoughts into consideration (38%, 9/24).

4) Qualities of mental health professionals

Young people were able to reflect upon the positive attributes of those mental health professionals that they had met. Young people had experienced ‘being listened to’ (68%), ‘being taken seriously’ (60%), ‘being believed in’ (56%), ‘friendliness’ (52%), ‘being seen as an individual’ (44%), ‘having someone interested in what they had to say’ (44%), ‘kindness (44%) and having someone who was ‘hopeful about their future’ (40%). Again, there was a very wide spread of qualities that they had found helpful.

They were also asked to think about the unhelpful and negative attributes that they had at times been exposed to in certain mental health professionals. The most prevalent response was that they had not always felt ‘understood’ (62%) whilst others did not feel ‘listened to’ (54%), or did not feel confident that the professional was sure what to do (50%), were made to feel ‘not normal’ (50%) and did not feel that the professional had not given ‘helpful’ responses (46%) to questions. Again, there was a diverse set of responses.

The parental survey tapped into how parents felt they were treated by the services. In many cases, the responses were positive: 74% felt they were seen to be ‘supportive’, 57% ‘caring’, 48% ‘interested’, 39% ‘understanding’, and 39% ‘determined’. At the other end of the spectrum, others felt they were perceived more negatively: 35% felt they were seen to be ‘demanding’, 30% ‘controlling’, 30% ‘annoying’, 30% ‘challenging’, 22% ‘interfering’, and 35% ‘demanding’.

Within the qualitative part of the survey, a very mixed picture was also evident but there was a common perception amongst a number of participants that they felt excluded and disenfranchised from their child’s treatment.

*‘I feel like I’m being left out. I have my son in an inpatient mental health facility and I don’t know what they are thinking about things on a daily basis. They don’t give me updates unless I ask.’*

Parents of young people had many thoughts about how such a service for young people should be designed. Many parents talked about the service not being overly dependent on drugs and one where a more eclectic and holistic approach was offered including counselling, peer groups, meditation, drug information sharing and alternative educational opportunities. One parent wrote of a wish to find *‘a place where young people can talk about what’s happening to them, their experiences and how they cope’.* Other comments from parents were far more focused on removing the stigma that has been so intrinsically part of their child’s problem, seeking *‘a centre where you can go and feel “normal”’* or *‘one that normalises the phenomenon. One that does not immediately rush to mental illness. One that can be presented to all children at school without stigmatizing any child.’*

Young people themselves had thoughts that echoed the parents’ perceptions, with many focusing on less reliance upon medication, a greater openness for sharing information and transparency amongst professionals. A large number of young people wanted to feel less judged and pathologised. One wrote: *‘It’s important for mental health care workers to see us as fully human and just as deserving of respect as them. Most of them don’t’.* Another wrote about her wish for *‘a service that doesn’t make you feel like everything is lost and you will have to be medicated for life, if not, your problem will become worse.’* Several of the young people wrote about it being '*hard to explain to those who aren’t in the same situation’.* They hoped for services in which they could link up with other young people who had experiences similar to their own.

## Discussion

The aim of this research was to explore the experiences of young people who hear voices and their families regarding mental health professionals and the services they provide. From the interviews, four superordinate themes arose: The first theme was the struggle to understand the hearing voices phenomenon stemming from what was described as a lack of information available. Neither parents nor young people felt that they had any satisfactory answers, leaving them feeling overwhelmed, lost and confused. Secondly, all participants felt that they were sometimes fighting a battle with the Mental Health Services for time and support. Parents and young people felt their voices were not heard and that there was a lack of connection between the service providers and receivers. The third theme that arose was the idea that young people who hear voices and their parents are both ‘stuck in a limbo’ because they cannot get the services and support the feel they need. The last theme described how both parents and young people wished for a more holistic approach from mental health services and professionals that would above all make them feel cared for, understood and believed and provide them with a space to share their vulnerable experiences.

The findings from the second part of this study partially confirmed the salience of these themes to other young people who hear voices and their parents; although it was clear that some families had more positive experiences of CAMHS. Like the interviewees, the vast majority of young people and parents surveyed had trouble accessing ‘useful’ information to aid their understanding of voice hearing, and like the interviewees, over half of parents and young voices hearers who took part in the survey described themselves as feeling ‘lost’. A large proportion of these parents and voice hearers reported that they found accessing help difficult, and almost half of the children thought that professionals had not considered their ideas, wishes and accounts ‘much’ or ‘at all’. Similarly, within the qualitative section of the parental survey, themes once again emerged pertaining to the wish to receive more holistic care and for approaches that would normalise the experience of voice hearing. Many more feelings were reported in the survey than had emerged from the interviews: feelings such as anxiety and stress were found in over half of young voices hearers and parents, with over half of the young voice hearers also reporting feeling lonely and crazy.

In line with results by Coffey and Hewitt [15], who found incongruity between help sought and help provided between voice hearers and community mental health nurses, this research suggests that young voice hearers may often face a similar experience in CAMHS. In particular, this research brings the focus onto young people and their families, who sometimes feel that mental health services are not adequately meeting their needs. The review of literature also suggested a lack of child-focused information and services in this field, which was voiced as a concern among participants in this study.

In addition, the young voice hearers and their families who took part in our study raised questions about the utility of the medical approach itself and expressed a wish for approaches that would help the child and family cope not just psychologically, but socially as well. Such findings sit uneasily alongside previous research that found biomedical models of voice-hearing were more likely to be validated by professionals than alternative explanations [16]. It may be that there is a need to make help accessible outside the mental health setting, for example in the form of peer support groups, which most participants in this study found extremely supportive. Previous research by Newton, Larkin, Melhuish and Wykes [24], investigating the experience of group-CBT amongst young people experiencing distressing auditory hallucinations, likewise found a cyclical relationship between the following key factors: the content of the hallucinated voices; the participants’ explanations for, and reactions to these voices; and thus, their ability to cope with them. They concluded, “'voices groups are appreciated by young people with auditory hallucinations, as sources of therapy, information, and support.” (p.127).

## Conclusions

Using a mixed-method approach, including both in-depth interviews and a web-based survey, this study confirms the struggle that many families of children who hear voices have, in trying to access appropriate support. At the same time, this research further extends our understanding of young people who hear voices and their families from a psychological to a social context as well. We have found that young people and their families found accessible information on voice-hearing hard to come by, that families sometimes ‘battle with the mental health services’, and that the gap between the actual and ‘ideal mental health service’ may have an important role to play in shaping their experience.

This study has examined the experience of a group of vulnerable young people and their families, whose voices have had little space in the professional literature to date. By combining a small-scale qualitative project with a larger questionnaire, we have tried to explore this potentially destabilising and overwhelming experience in a form that can be helpful in terms of indicating what changes could be brought about in the system to help these families feel understood and helped.

However this study has some limitations. Firstly, the sample size for study one was small, and although the relevance of the themes identified was tested on a larger sample in study two, caution about the generalizability of these findings is still called for. As they were self-selecting, neither the two families who took part in the first study, nor those who chose to access the online survey in stage two of our study can be considered 'representative' of all voice hearers and their families. Similarly, it is also difficult to generalise this research to other ethnic groups, as ethnicity was not captured in the online survey and both parent–child dyads that were interviewed were Caucasian.

Notwithstanding these limitations, this study provides us with the first experiences of engaging with mental health services from both the perspectives of young voice hearers and their parents; and therefore provides important indications of the key challenges experienced by these families in accessing support. In particular, this study points to a strong need to develop services that engage with families in a supportive and collaborative way, using age-appropriate language and explanatory models that go beyond the biomedical one, which was not always found to be helpful by the families and young voice hearers who took part in this study.

Considering these findings, it would be beneficial for staff who work in CAMHS and early intervention teams to receive further specialised training in how to speak with young people about the voices they hear and approach voices in a way that is meaningful to both the family and the individual. Additionally, young voice hearers and their families may also benefit from hearing voice peer support groups that could form part of a ‘normalising’ and ‘holistic care’ package that could be facilitated by CAMHS professionals and, ideally, experts with experience of voice hearing.

## Additional files

Additional file 1:
**Questionnaire completed by young people.** Description of data: Copy of questionnaire completed by young people. Additional file 2:
**Questionnaire completed by parents/guardians.** Description of data: Copy of questionnaire completed by parents/guardians.
