# Supplementary material for: The experience of engaging with mental health services among young people who hear voices and their families: a mixed methods exploratory study
Source: BMC Health Serv Res. 2014 Nov 5;14:527. doi: 10.1186/s12913-014-0527-z (PMC4256811; doi:10.1186/s12913-014-0527-z)
Supplement: Additional file 1: — Questionnaire completed by young people. Description of data: Copy of questionnaire completed by young people. [file 12913_2014_527_MOESM1_ESM.pdf]

Introduction

Thank you for taking the time to look at this survey. My name is Prerna Kapur. I am a student at the Anna Freud Centre and University College London. I've just done a small study into the experiences of young people who hear voices and their families of mental health services and professionals. I'm interested to find out whether the experiences of the young people I spoke to in my research are similar to, or different from, other young people. This survey is anonymous, but the results will be used in my write up. I'll be sharing the results on the Voice Collective website too. It would only take 10-12 minutes to fill out this short survey and I would really appreciate your input.

Contents

Page 1: More about the research
Page 2: What I found in the interviews (for you to have a look at)
Page 3: The survey

More about the research

This research is aimed at understanding the experiences of young people who hear voices and their families of mental health services and professionals. This study consisted of 4 participants, 2 young people and 2 parents. The interviews consisted of questions asking the young people a little about themselves and their experience of hearing voices. It also had some questions about any help that they have had for dealing with these voices, the help that they are currently getting and what they feel about it. We then tried to study the experiences of all 4 participants together and found some common feelings and experiences that they all seem to have. You can read more about these on the next page.

This research has got ethical approval from UCL. If you'd like further information or would like to get in touch for any other reason, you can contact me or either of my supervisors, Nick Midgley and Saul Hillman at the address and number mentioned below:

The Anna Freud Centre,
12 Maresfield Gardens,
London
NW3 5SD.
Tel: 020 7794 2313

Summary of Research findings

Following are the themes through which we have tried to capture, to the best of our ability, the essence of the experiences of the participants who took part in this research:

1. The struggle to understand the hearing voices phenomenon
Both young people and their parents who took part in this study spoke about their struggle with finding an explanation for the 'hearing voices' phenomenon. They vividly describe the lack of information available, which tends to leave them anxious about what is happening to the young person. There are no definite answers, which may result in them feeling lost and confused. Amidst this confusion, the parents go back to the one thing that they are sure about i.e. their relationship with their child and their knowledge of them. The young people often feel anxiety stemming from a lack of understanding about what is going on for them.

2. Battling with the Mental Health Services
Among the participants in this study there was skepticism about the available mental health support expressed both by young people and their parents. For the young people, there seemed to be a continuous conflict between what they needed and hoped to receive as a form of support, on the one hand, and what they were actually provided by CAMHS, on the other. For the parents, the conflict seemed to lie in being seen as both a supporter outside the mental health service system, however an unwanted intrusion within the system. Both young people and their parents felt a definite barrier between themselves and mental health professionals and felt as if their voices are not being heard. There was also a feeling of being judged and stereotyped and not being believed. For the parents, the struggle

with mental health services was often linked to them having to be resourceful themselves.

3. ‘Stuck in a limbo’
Young people and their parents seemed to feel as if they were living in a continuous state of frustration due to the slow treatment process at CAMHS and the inconsistency in the availability of services. There was a feeling of helplessness and loss of control. None of the participants in this study seemed very hopeful about their treatment. This state made the young people often feel unwanted, unworthy and as if they and their needs were a ‘waste of time’.

4. Wish for a holistic approach
Both young people and parents hoped for a more all-inclusive approach that would provide a space to share and express themselves without feeling judged. They hoped for an approach that is child-centred and uses a language that the young person can understand, thus not leaving the young person overwhelmed. They also looked for alternative help, i.e. other than medication, including greater community support and empathic understanding. At the same time, those professionals who were perceived as caring, understanding, believing and going that extra mile were the ones each of the interviewees valued. These appear to be islands/sanctuaries within a sea of what seemed to be a very overwhelming experience in which they felt misunderstood and disbelieved.

Please move to the next page to fill out a short survey about this research and your own personal experience with mental health services and professionals

*1. Please enter the following details

Age (in years) ________

Gender (Male/Female) ________

Country ________

2. Whilst some people feel very sure about the reasons they hear voices, other feel unsure about where they come from. Do you feel you know why you hear voices?

- ○ Yes, I know why I hear voices
- ○ I mostly know why I hear voices, but have some doubts
- ○ I some ideas about why I hear voices, but I don't know which is right
- ○ I don't know why I hear voices

3. How have you found accessing useful information to help you understand your experiences of hearing voices?

- ○ Very easy
- ○ Quite easy
- ○ Quite difficult
- ○ Very difficult

4. How do you feel about being a young person who hears voices? (You can choose more than one option)

- ☐ Proud
- ☐ Anxious
- ☐ Stressed Out
- ☐ Confused
- ☐ Special
- ☐ Creative
- ☐ Scared
- ☐ Lonely
- ☐ Crazy
- ☐ Left Out
- ☐ Angry
- ☐ Normal
- ☐ Calm
- ☐ Confident
- ☐ Included
- ☐ Lost

5. How easy has it been to access the kind of help you need in the mental health services?

- ○ Very Easy
- ○ Quite Easy
- ○ Quite difficult
- ○ Very difficult
- ○ I have never tried to get help from the mental health services

6. In general, how much do you think your ideas, wishes and views are taken into account by the professionals involved in your care?

- ○ A lot
- ○ A little
- ○ Not much
- ○ Not at all

7. When thinking about the most helpful mental health professionals that you've met, what was best about them? (You can choose more than one option)

- ☐ Approachable
- ☐ Friendly
- ☐ They listen to me
- ☐ They understand me
- ☐ They see me as an individual
- ☐ They are interested in what I say
- ☐ They are confident
- ☐ They are hopeful about my future
- ☐ They have good ideas
- ☐ They go 'the extra mile' to help
- ☐ They are kind
- ☐ They help me feel normal
- ☐ They see me as an equal
- ☐ They believed me
- ☐ They took me seriously

8. Thinking about the least helpful mental health professionals you've met, what was worst about them? (You can choose more than one option)

- ☐ Unapproachable
- ☐ Unfriendly
- ☐ They didn't listen to me
- ☐ They seemed unsure what to do
- ☐ They made me feel not-normal
- ☐ They made me feel unimportant
- ☐ They didn’t believe me
- ☐ They were resigned about my future
- ☐ They didn’t understand me
- ☐ They didn’t have very helpful ideas
- ☐ They did not want to make an extra effort to help
- ☐ They did not take me seriously

9. If you could design a mental health service for young people like you, what would it be like (you can tell us about anything that is important to you - you don't have to be realistic)

10. A. Do you think your own personal experience is similar or different from the findings of this Research?
B. Is there anything else you'd like to tell us about your experiences of mental health professionals?
Please feel free to use this space to share with us your personal experience.
