# Supplementary material for: The experience of engaging with mental health services among young people who hear voices and their families: a mixed methods exploratory study
Source: BMC Health Serv Res. 2014 Nov 5;14:527. doi: 10.1186/s12913-014-0527-z (PMC4256811; doi:10.1186/s12913-014-0527-z)
Supplement: Additional file 2: — Questionnaire completed by parents/guardians. Description of data: Copy of questionnaire completed by parents/guardians. [file 12913_2014_527_MOESM2_ESM.pdf]

## Introduction

Thank you for taking the time to look at this survey. My name is Prerna Kapur. I am a student at the Anna Freud Centre and University College London. I've just done a small study into the experiences of young people who hear voices and their families of mental health services and professionals. I'm interested to find out whether the experiences of the parents I spoke to in my research are similar to, or different from, other paremts. This survey is anonymous, but the results will be used in my write up. I'll be sharing the results on the Voice Collective website too. It would only take 10-12 minutes to fill out this short survey and I would really appreciate your input

Contents

Page 1: More about the research
Page 2: What I found in the interviews (for you to have a look at)
Page 3: The survey

## More about the research

This research is aimed at understanding the experiences of young people who hear voices and their families of mental health services and professionals. This study consisted of 4 participants, 2 young people and 2 parents. The interviews for parents asked about their experience of having a child who hears voices. They were also asked questions regarding previous contact and help that they have had with mental health services. Further questions focused on what support structures they use to cope with the problem, and the expectations and anxieties that they may have related to the mental health services and professionals they've had contact with. We then tried to study the experiences of all 4 participants together and found some common feelings and experiences that they all seem to have. You can read more about these on the next page.

This research has got ethical approval from UCL. If you'd like further information or would like to get in touch for any other reason, you can contact me or either of my supervisors, Nick Midgley and Saul Hillman at the address and number mentioned below:

The Anna Freud Centre,
12 Maresfield Gardens,
London
NW3 5SD.
Tel: 020 7794 2313

## Summary of Research findings

Following are the themes through which we have tried to capture, to the best of our ability, the essence of the experiences of the participants who took part in this research:

1. The struggle to understand the hearing voices phenomenon
Both young people and their parents who took part in this study spoke about their struggle with finding an explanation for the 'hearing voices' phenomenon. They vividly describe the lack of information available, which tends to leave them anxious about what is happening to the young person. There are no definite answers, which may result in them feeling lost and confused. Amidst this confusion, the parents go back to the one thing that they are sure about i.e. their relationship with their child and their knowledge of them. The young people often feel anxiety stemming from a lack of understanding about what is going on for them.

2. Battling with the Mental Health Services
Among the participants in this study there was skepticism about the available mental health support expressed both by young people and their parents. For the young people, there seemed to be a continuous conflict between what they needed and hoped to receive as a form of support, on the one hand, and what they were actually provided by CAMHS, on the other. For the parents, the conflict seemed to lie in being seen as both a supporter outside the mental health service system, however an unwanted intrusion within the system. Both young people and their parents felt a definite barrier between themselves and mental health professionals and felt as if their voices are not being

heard. There was also a feeling of being judged and stereotyped and not being believed. For the parents, the struggle with mental health services was often linked to them having to be resourceful themselves.

3. ‘Stuck in a limbo’
Young people and their parents seemed to feel as if they were living in a continuous state of frustration due to the slow treatment process at CAMHS and the inconsistency in the availability of services. There was a feeling of helplessness and loss of control. None of the participants in this study seemed very hopeful about their treatment. This state made the young people often feel unwanted, unworthy and as if they and their needs were a ‘waste of time’.

4. Wish for a holistic approach
Both young people and parents hoped for a more all-inclusive approach that would provide a space to share and express themselves without feeling judged. They hoped for an approach that is child-centred and uses a language that the young person can understand, thus not leaving the young person overwhelmed. They also looked for alternative help, i.e. other than medication, including greater community support and empathic understanding. At the same time, those professionals who were perceived as caring, understanding, believing and going that extra mile were the ones each of the interviewees valued. These appear to be islands/sanctuaries within a sea of what seemed to be a very overwhelming experience in which they felt misunderstood and disbelieved.

Please move to the next page to fill out a short survey about this research and your own personal experience with mental health services and professionals

## *1. Please enter the following details

Age (in years) ______

Gender (Male/Female) ______

Country ______

Child's Age (in years) ______

Child's Gender (Male/Female) ______

## 2. Whilst some people feel very sure about the reasons their child hears voices, others feel unsure about where they come from. Do you feel you know why your child hears voices?

- ○ Yes, I know why my child hears voices
- ○ I mostly know why my child hears voices, but I have some doubts
- ○ I have some ideas about why my child hears voices, but I don't know which is right
- ○ I don't know why my child hears voices

## 3. How have you found accessing useful information to help you understand your child’s experiences of hearing voices?

- ○ Very easy
- ○ Quite easy
- ○ Quite difficult
- ○ Very difficult

**4. How do you feel about being a parent of a young person who hears voices? (You can choose more than one option)**

- ☐ Proud
- ☐ Anxious
- ☐ Stressed Out
- ☐ Confused
- ☐ Scared
- ☐ Lonely
- ☐ Left Out
- ☐ Angry
- ☐ Normal
- ☐ Calm
- ☐ Confident
- ☐ Included
- ☐ Lost
- ☐ Blamed
- ☐ Doubted

**5. How easy has it been to access the kind of help your child needs in the mental health services?**

- ○ Very Easy
- ○ Quite Easy
- ○ Quite difficult
- ○ Very difficult
- ○ I have never tried to get help for my child from the mental health services

**6. In general, how much do you think your ideas, wishes and views are taken into account by the professionals involved in your child's care?**

- ○ A lot
- ○ A little
- ○ Not much
- ○ Not at all

**7. How do you feel you as a parent are seen by mental health services? (You can choose more than one option)**

- ☐ Interfering
- ☐ Controlling
- ☐ Determined
- ☐ Supportive
- ☐ Caring
- ☐ Understanding
- ☐ Valuable
- ☐ An asset
- ☐ A problem
- ☐ Challenging
- ☐ Creative
- ☐ Annoying
- ☐ Nosy
- ☐ Interested
- ☐ Irrelevant
- ☐ Demanding

**8. If you could design a mental health service for young people like your child, what would it be like (you can tell us about anything that is important to you - you don't have to be realistic)**

**9. Do you think your own personal experience is reflected from the findings of this Research? Please feel free to give your comments and feedback.**

**10. Is there anything else you'd like to tell us about your experiences of mental health professionals?**
**Please feel free to use this space to share with us your personal experience.**
